# Exploring complexity of class-A Beta-lactamase family using physiochemical-based multiplex networks

**DOI:** 10.1038/s41598-023-48128-y

**Published:** 2023-11-23

**Authors:** Pradeep Bhadola, Nivedita Deo

**Affiliations:** 1 Centre for Theoretical Physics & Natural Philosophy, Mahidol University, Nakhonsawan Campus, Phayuha Khiri, NakhonSawan, 60130 Thailand; 2 Department of Physics and Astrophysics, University of Delhi, Delhi, 110007 India

**Keywords:** Biological physics, Computational biology and bioinformatics

## Abstract

The Beta-lactamase protein family is vital in countering Beta-lactam antibiotics, a widely used antimicrobial. To enhance our understanding of this family, we adopted a novel approach employing a multiplex network representation of its multiple sequence alignment. Each network layer, derived from the physiochemical properties of amino acids, unveils distinct insights into the intricate interactions among nodes, thereby enabling the identification of key motifs. Nodes with identical property signs tend to aggregate, providing evidence of the presence of consequential functional and evolutionary constraints shaping the Beta-lactamase family. We further investigate the distribution of evolutionary links across various layers. We observe that polarity manifests the highest number of unique links at lower thresholds, followed by hydrophobicity and polarizability, wherein hydrophobicity exerts dominance at higher thresholds. Further, the combinations of polarizability and volume, exhibit multiple simultaneous connections at all thresholds. The combination of hydrophobicity, polarizability, and volume uncovers shared links exclusive to these layers, implying substantial evolutionary impacts that may have functional or structural implications. By assessing the multi-degree of nodes, we unveil the hierarchical influence of properties at each position, identifying crucial properties responsible for the protein’s functionality and providing valuable insights into potential targets for modulating enzymatic activity.

## Introduction

Protein’s ability to perform biochemical functions relies on the interactions and dynamics of their constituent amino acids, forming a molecular network within living cells that governs various cellular processes. Understanding this complex network and identifying the amino acid residues and interactions responsible for protein structure and function is a fundamental challenge in molecular biophysics^1^. Co-evolution studies within protein families have seen a significant rise, serving as the foundation for numerous influential studies^2–10^. While many methods treat protein sequences as mere strings of characters and derive inferences from amino acid distributions, entropy, or mutual information^3–9^, there are other approaches such as evolutionary rate methods, ensemble approaches, structure-based function prediction, machine learning methods^3,4,9,10^, which do not entirely rely on amino acid frequencies but require additional information about the structure or evolutionary history. Most of these methods fail to incorporate the physical, chemical, and biological properties of amino acids leading to incomplete or false-positive results whereas it has been established that evolution and the function of an amino acid inside a protein sequence greatly depends on its physiochemical properties^11–13^. The physiochemical nature of the constituent amino acids is responsible for guiding the protein to its native state^14^. Additionally, in most amino acid co-evolution studies, an underlying assumption is that only one type of interaction exists between any two positions represented as the average co-evolutionary interaction between the positions. This assumption ignores the possibility of multiple interactions or relationships between different positions.

The focus of this research lies in investigating the intricate network of amino acids, wherein positions within a sequence engage in multiple interactions with other positions. Such a system, known as a multiplex network, is characterized by the presence of shared multiple interactions among the same entities^15^. Within a multiplex network, each layer represents a unique relationship among a common set of nodes. An illustrative example of a multiplex network is a social network, where individuals interact through various platforms such as Twitter and Facebook, depicted as different network layers. Multiplex networks serve as a valuable tool for modeling and analyzing complex systems, providing a more comprehensive understanding of their structure and functionality^15–18^. Real-world systems, such as social systems, and biological systems, can be effectively represented and studied using multiplex network approaches^17,18^. By employing multiplex network analysis, we can uncover new insights into the organization and dynamics of complex systems, identify crucial nodes and communities, and gain a deeper understanding of their functioning^18–20^. We propose a multiplex network for the class-A $\beta$-lactamase enzyme family where the positions can have multiple interactions with other position, where these interactions are expressed as a distinct layer of the network and depend on the physiochemical properties of the amino acids.

## System and data

The focus of this study centers around the class A $\beta$-lactamase enzyme family, which plays a crucial role as the primary defense mechanism of bacteria against $\beta$ -lactam antibiotics such as penicillin and cephamycins^21^. The remarkable ability of the $\beta$ -lactamase enzyme to alter its substrate specificity with just a few mutations^22,23^ poses a significant challenge to the future effectiveness of antibiotics. Therefore, a comprehensive understanding of the enzyme’s structure and function is of utmost importance, as it will facilitate the development of more efficacious drugs^24^. To conduct our analysis, we employed a dataset consisting of 5447 protein sequences from the Interpro entry (IPR000871) in the Interpro protein data bank^25^ pertaining to the class A $\beta$-lactamase family. Raw protein sequences may contain phylogenetic effects and historical noise that can significantly impact the analysis and yield spurious results. To address these factors, we implemented a rigorous filtering process to eliminate highly similar sequences with a similarity greater than $90\%$, resulting in a set of 559 unique sequences. Subsequently, after cleaning these sequences were aligned using a multiple sequence alignment (MSA) comprising 248 columns representing amino acid positions in proteins [Details of the filtering process is given in Supplementary Material].

**Figure 1:**
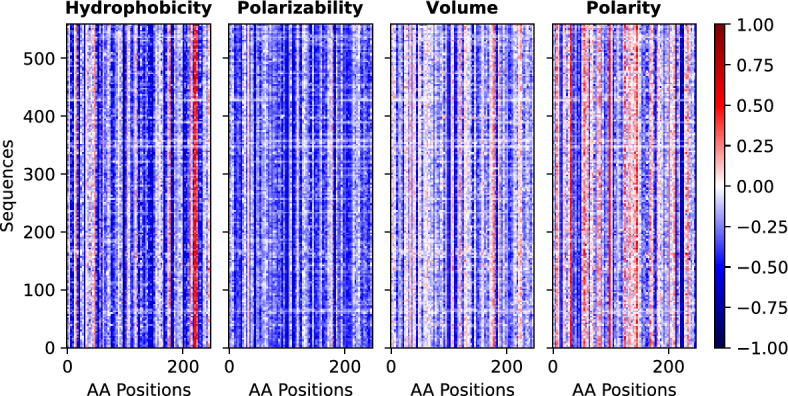
Datamatrix for class-A $\beta$-lactamase all 4 properties.

In order to incorporate various physical, chemical, and biological properties of amino acids, we used four distinct physiochemical properties: hydrophobicity, polarizability parameter, polarity, and Vander Waal’s volume along with the MSA. These properties were first extracted from various sources^26–28^ and then re-scaled to a range of $-1$ to 1, where a value of 1 indicates the highest value of a specific property among all amino acids, while $-1$ represents the lowest value of the same property. These re-scaled physiochemical properties were then incorporated into the multiple sequence alignment (MSA) to convert the two-dimensional MSA into a three-dimensional data matrix denoted as $D^{\alpha }_{s}(i)$, where $\alpha (= {1 \cdots 4)}$ denotes different physiochemical properties, *i* represents the position of amino acids (column) and *s* represents the protein sequence (row). For the class-A $\beta$-lactamase family the dimensions of the data matrix for each property is $S \times L$ with $S = 559$ as the number of sequences and $L=248$ as the number of positions in the MSA. Figure 1 visually represents the data matrix for the class-A $\beta$-lactamase family using four different physiochemical properties. Notably, the data matrix changes with properties, even though it is derived from the same MSA, suggesting that each property provides unique and valuable information.

## Weighted multiplex threshold network

For each physiochemical property, denoted as $\alpha$, we calculate the Pearson’s correlation coefficient between different positions of the data matrix $D^{\alpha }$. The correlation coefficient $C^{\alpha }_{ij}$ between position *i* and *j* of the data matrix $D^{\alpha }$ for a fixed $\alpha$ is given by

1 $$\begin{aligned} C^{\alpha }_{ij} = \frac{\big<d^{\alpha }_i- \big<d^{\alpha }_i\big>\big>.\big< d^{\alpha }_j-\big <d^{\alpha }_j\big>\big >}{ \sigma (d^{\alpha }_i) \sigma (d^{\alpha }_j)}. \end{aligned}$$

Here, $d^{\alpha }_i$ represents the $i$th column of the data matrix $D^{\alpha }$, $\sigma (d^{\alpha }_i)$ represents the standard deviation of $i$th column of the data matrix $D^{\alpha }$ and $<\cdots>$ denotes the average over the proteins sequences^13^. These correlation coefficients are utilized to construct a network that represents the evolutionary interaction for the class-A $\beta$-lactamase enzyme family. However, due to the multiple relationships among the nodes (positions) in the network resulting from different physiochemical properties, a simple network representation is insufficient. To capture the multiple interactions among the nodes effectively, we employ a multiplex network approach^29^.

A multiplex network consists of layers, where the same set of nodes exists in different layers and each layer represents a different form of interaction or communication among entities.

A multiplex network is a set of networks $G^{\alpha } =(N,E^{\alpha })$ arranged in layers, with $\alpha =1,\ldots,L$ with *L* as the number of layers. The set of nodes *N* is the same in each layer whereas the set of edges $E^{\alpha }$ is layer dependent. For the class-A $\beta$-lactamase enzyme family, each multiplex network layer $G^{\alpha }(N, E^{\alpha })$, is characterized by $\alpha = 1,\ldots, L$ corresponds to a specific physiochemical property, the nodes (*N*) represents the positions in the MSA and a edge $E^{\alpha }_{i,j}$ between two nodes *i* and *j* is given by

2 $$\begin{aligned} E^{\alpha }_{i,j} = \left\{ \begin{array}{ll} |C^{\alpha }_{ij}| &\quad {\text{if}} \; i\ne j, |C^{\alpha }_{ij}|\ge \theta \\ 0 &\quad {\text{otherwise}}, \end{array} \right. \end{aligned}$$

where $\theta$ is the threshold value and $C^{\alpha }_{ij}$ is the Pearson correlation coefficient between position *i* and *j* for a physiochemical property ($\alpha$). The threshold serves as a cutoff value for deciding which correlations are significant enough to be represented in the threshold network. Correlation coefficients that meet or exceed this threshold value are considered strong correlations and are included in the network, while those below the threshold are excluded. In the multiplex network, the interlayer links connect a given node to its counterpart nodes in the rest of the layers. The intra-layer links physically represent the extent of the evolutionary interaction between two positions *i* and *j* in a layer. In present context, the evolutionary interaction refers to the co-evolutionary relationships among amino acids physicochemical properties within the class-A $\beta$-lactamase enzyme family that are conserved throughout the evolutionary history of the protein. We aim to comprehend how these physiochemical properties have collectively evolved over time, providing insights into their functional and structural roles. The links that are preserved at higher thresholds indicate the interactions that have been conserved throughout the evolutionary history of the protein. A link is present between two positions if the absolute correlation strength between them is greater than the threshold for the given physiochemical property. Since 4 different physiochemical properties are used, the multiplex network has 4 different layers ($L=4$), each layer representing one property. The complete multiple network is denoted as $\vec {G} = (G^1,G^2,G^3, G^{4})$, where $G^\alpha$ is the network corresponding to the property $\alpha$. Each physiochemical property contributes a network layer with the same set of nodes but with a different set of connections. The changes in the network with the number of sequences used for analysis is given in the Supplementary Material Tables 1 and 2.

**Figure 2:**
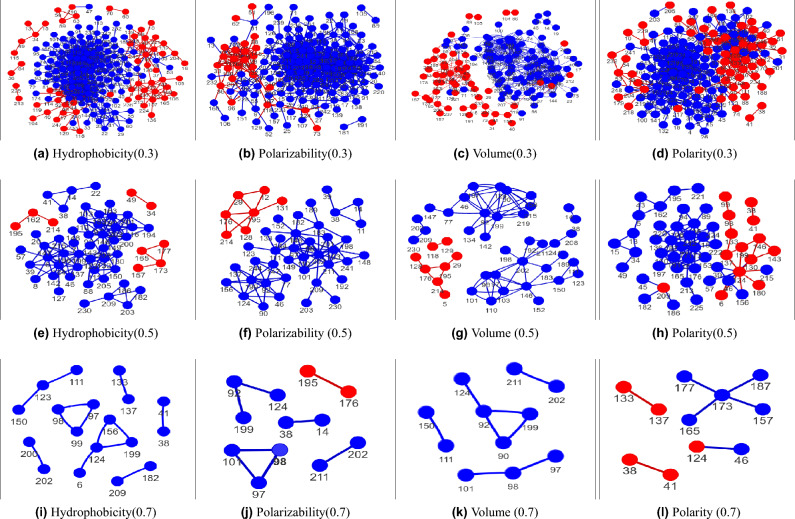
Layers of the multiplex network at different thresholds (cut-off) on correlation coefficients [0.3 (**a**-**d**), 0.5 (**e**-**h**), 0.7 (**i**-**l**)] showing only nodes with non-zero connections for clarity. The blue color indicates a negative average value of the physiochemical property at that position whereas the red color represents the positive average value. Node numbering follows the filtered MSA, differing from the Ambler numbering scheme.

By changing the threshold, the multiplex network structure changes.An increase in threshold results in the isolation of nodes within the network layers whose interaction strength with other nodes is smaller than the threshold value for the given physiochemical property. Higher thresholds lead to sparser networks with stronger interactions among nodes within each layer. Figure 2 shows the different network layers at threshold values of 0.3, 0.5, and 0.7, respectively, with only nodes having non-zero connections shown for clarity. The nodes are colored by the average value of the physiochemical property at that position. Mathematically, the average value at position *i* for property $\alpha$ is given by $\big <d^{\alpha }_i\big>$ where $d^{\alpha }_i$ represents the $i$th column of the data matrix $D^{\alpha }$ and $\big < \cdots \big>$ is the average over the sequences. If the average value of a physiochemical property $\alpha$ at a position is positive then the node is colored red and a blue-colored node implies a negative value of the physiochemical value at that position. By analyzing each layer of the network, it is evident that the positions tend to interact and connect with the other positions having similar values of the physiochemical properties. We observe the formation of patterns in each layer based on the average value of physiochemical properties, where for each layer the blue nodes tend to have higher connections with blue nodes whereas red nodes tend to connect with red nodes. At higher thresholds (Figure 2), the nodes are completely separated into components based on the average value of the physiochemical property. This implies that the interaction between positions depends highly on the value of the physiochemical property. There are only a few interactions between the two groups (red and blue) of node, most of the interactions are among the members of the group.

### Filtering edges, information loss, and randomness

Threshold networks are often plagued with random and statistical noise at low thresholds, which can hinder their accuracy. As the threshold increases, the network becomes sparser by removing spurious and weak edges. However, this deletion of edges with smaller weights, or correlation coefficients, comes at the cost of information loss. Conversely, a smaller threshold value results in a larger number of random and spurious edges. To address these challenges and determine the optimal threshold value that minimizes spurious connections while retaining maximum information, we employ the random matrix theory (RMT). Random matrix theory, extensively used in diverse fields such as the study of RNA structures^30,31^, proteins^13^, stock market^32^, wireless communication^33^ and many more, provides a framework for analyzing correlation matrices and filtering out noise. Specifically, we utilize a class of matrices called Wishart matrices, which have been extensively used for information filtering of correlation matrices to separate the relevant information from the noise^13,32,34^. We used Wishart matrices to establish the lower bounds for random thresholds. By numerically simulating the bounds of correlation coefficients using an ensemble of Wishart matrices (with dimensions the same as the system), we obtain a reliable approximation for the lower threshold that effectively reduces noise. To validate the effectiveness of our approach, we compare the lower bounds obtained from Wishart matrices with those obtained by shuffling the original data. Remarkably, both methods yield nearly identical results, affirming the reliability of our findings.

For our dataset, the bounds of average random correlations $C^{rand}_{ij}$ are given by $-0.2<C^{rand}_{ij} <0.2$. Accordingly, we classify the threshold values into three distinct regions. The first region corresponds to $\theta <0.2$, where randomness dominates, making it challenging to determine genuine interactions amidst spurious ones. As the threshold value surpasses 0.2 ($\theta >0.2$), we enter the second region, where noise progressively decreases, allowing the emergence of actual links. Notably, a small band exists just beyond the 0.2 threshold, indicating that randomness still contributes significantly to the network links. To achieve a significant reduction in noise, we find that a threshold value of $\theta >0.3$ is effective for the system. At higher thresholds, we observe links that reflect strong co-evolution between positions during the course of evolution. It is worth noting that many of these links are crucial for the enzymatic activity and structural stability of the protein family.

### Properties of individual layer

We conducted an extensive analysis of the topological properties of each individual layer at varying thresholds, with detailed results presented in Table. 1. It is crucial to emphasize that these topological properties are influenced by the specific layer being analyzed as well as the chosen threshold values. In the context of the protein threshold network, a node’s degree signifies the number of evolutionary interactions that a position maintains with other positions within the protein sequence. Meanwhile, the threshold determines the minimum required strength for these interactions to be considered valid. Nodes with higher degrees represent a highly interacting position. When observing the variation of degree with thresholds, we noted that below the 0.25 threshold, all nodes exhibited significantly high degrees, primarily due to statistical noise. In this region, it is difficult to separate random noise from the system information. However, randomness decreases with the increase in threshold, and at higher thresholds, the nodes with non-zero degrees emerge as vital contributors to the enzyme’s functionality. At threshold 0.8, only a select few nodes ( 38, 41, 97, 98, 99, 200, 202, 211) show interaction in at least one layer of the multiplex network. These nodes correspond with the following Ambler numbers (AN) **70, 73, 130, 131, 132, 234, 236, 247** [All Ambler number (AN) will be given in boldface]. A comprehensive mapping between the nodes in the network and the Ambler number is provided in the Supplementary Material. Among these, position 38 serves as the primary catalytic residue, while positions 41, 97, 98, 99, 200, and 202 are also associated with catalytic functions^35,36^. All these positions are present in the hydrophobicity layer, except position 211 appears in polarizability found to be involved in the acylation mechanism^37^.

**Table 1:** Topological Properties of the class-A $\beta$-lactamase family such as density, number of edges, average clustering ($C_{avg}$), average degree ($K_{avg}$), maximum degree ($K_{max}$), size of largest component ($N_{comp}$), average path length ($L_{avg}$), Radius (*R*), and Average Eccentricity ($\varepsilon$) at different threshold $\theta$.

$\theta$	Property	Density	Edges	$C_{avg}$	$K_{avg}$	$K_{max}$	$N_{comp}$	$L_{avg}$	*R*	$\varepsilon$
0.1	Hydrophobicity	0.2408	7365	0.519	59.39	129	248	1.87	3	3.00
	Polarizability	0.2854	8740	0.530	70.484	145	248	1.74	2	2.81
	Volume	0.2204	6753	0.478	54.459	114	248	1.85	2	2.99
	Polarity	0.2019	6185	0.472	49.879	88	248	1.89	3	3.04
0.3	Hydrophobicity	0.0275	842	0.388	6.790	52	177	3.72	5	7.78
	Polarizability	0.0298	914	0.339	7.371	55	168	3.37	5	6.59
	Volume	0.0209	639	0.278	5.153	36	143	4.398	7	8.61
	Polarity	0.0237	726	0.331	5.855	33	158	3.54	5	6.25
0.5	Hydrophobicity	0.0042	129	0.127	1.040	17	39	2.59	3	4.13
	Polarizability	0.0031	95	0.109	0.766	14	24	2.25	3	3.67
	Volume	0.0025	77	0.080	0.621	11	18	2.39	3	3.83
	Polarity	0.0030	92	0.093	0.742	15	22	2.10	3	3.77
0.7	Hydrophobicity	0.0004	13	0.022	0.104	3	4	1.33	1	1.75
	Polarizability	0.0003	8	0.012	0.065	2	3	1.33	1	1.67
	Volume	0.0002	8	0.009	0.064	3	4	1.33	1	1.75
	Polarity	0.0002	7	0.000	0.056	4	5	1.60	1	1.80

In our analysis, we have identified that a limited number of positions participate in evolutionary interactions within the network. For instance, at a threshold of 0.5, only 103 distinct positions (out of 248 positions) actively contribute in at least one layer of the multiplex network, with 145 positions either lacking interactions or displaying interaction strengths below 0.5. We identified 57 positions associated with hydrophobicity, 46 positions with polarizability, 44 positions with volume, and 53 positions with polarity, all exhibiting non-zero interactions, with most of the above positions showing interactions in more than one layer [See Supplementary Information Table 3]. Some specific positions stand out due to their remarkably high degrees compared to others. At 0.5 threshold, for hydrophobicity positions 6, 97, 99, 124, 146, 199, 200, 202, [**AN: 37, 130, 132, 157, 179, 233, 234, 236**], for polarizability positions 92, 146, 199, 202, 211, [** AN: 125,179, 233, 236, 247**] for volume positions 92, 199, 202 [**AN: 125, 233, 236**], and for polarity positions 124, 173, 177, 187 [**AN: 157, 207, 211, 221**] act as hubs with high degree exceeding 8 compared to other positions. Hubs identified by positions 6, 97, 99, 124, 146, 173, 199, 200, and 202 are essential positions for catalysis and/or substrate binding in class-A $\beta$-lactamases^36,38^, position 211 was found to be involved in the acylation mechanism^37^ while other positions 92 and 177 are the conserved residues^38^, position 187 was found to have interaction with ceftazidime and cefotaxime^36^.

Furthermore, increasing the threshold, led to the elimination of most of the nodes, retaining only those with strong evolutionary interactions. As discussed earlier, at 0.8 threshold only 8 out of 248 positions have non-zero interactions. These positions, especially in the hydrophobicity layer, coincide with a conserved motif for the protein family^35,36^, highlighting their crucial role in controlling the protein’s activity, often linked to catalytic or substrate binding positions^35,36^. At higher thresholds, nodes with high clustering coefficients form the core of important motifs within the enzyme family, particularly within the hydrophobicity layer, where a modular structure emerges due to collaborative functional interdependence during the evolutionary process. Physically, in the protein network, the nodes with high clustering coefficients indicate that they are surrounded by positions that have strong interactions with each other. Therefore, these nodes are part of the neighborhood that has highly interconnected neighbors with strong interaction, indicating a high possibility of functional and evolutionary constraints. These neighborhoods are sometimes part of the sector that forms the important regions in the proteins. For example, at 0.6 threshold positions with high clustering are identified as 46, 90, 97, 98, 101, 103, 105, 110, 111, 115, 123, 124, 149, 150, 157, 160, 182, 194, 199, 201, 203, 208, 209, 211 [**AN: 78, 123, 130, 131, 134, 136, 138, 143, 144, 148, 156, 157, 182, 183, 190, 193, 216, 228, 233, 234, 235, 237, 244, 245, 247**] [Supplementary Material Figure 3]. Some of these positions are catalytic and/or involved in substrate binding/activity 97, 98, 101, 103, 111, 123, 124, 199, 200, 201, 203^35,36^ whereas others are highly conserved positions 46, 90, 110, 149, 150, 157, 182, 209^35^, positions 208 is conserved as well as involved in stabilization of the initial enzyme-substrate complex^37^. Position 211 was found to be involved in the acylation mechanism^37^. However, four positions, namely 105, 115, 160, and 194, lack existing literature references for the class-A $\beta$-lactamase family. Interestingly, positions 105, 115, and 160 predominantly appear in the polarity layer, indicating their interactions involve polar forces, while position 194 seems to engage in hydrophobic interactions. These positions with high clustering coefficients are part of a neighborhood with extensive evolutionary connections. This suggests that they could hold significance within the family and might serve as promising targets for drug development. It is important to note that the observed clustering patterns are specific to each property, allowing for the identification of evolutionary interactions between positions for a specific property.

The presence of the modular structure in the network may be the outcome of the collaborative functional interdependence during the evolutionary process. The components of the network extracted at different thresholds have both structural and functional significance.

The class-A $\beta$-lactamase family contains four conserved motifs which are $^{38}SXXK^{41}$, $^{97}SDN^{99}$, $^{133}EXXLN^{137}$ and $^{200}KTG^{202}$ [**AN**:$^{70}SXXK^{73}$, $^{130}SDN^{132}$, $^{166}EXXLN^{170}$ **and** $^{234}KTG^{236}$] The graph-theoretical approach uniquely and distinctly extracts these motifs based on the hydrophobicity scale (at $\theta =$ 0.8), which also gives the interaction between positions. The strongest component $200-202$ is extracted at threshold 0.95 corresponds to the $^{200}KTG^{202}$ motif. According to graph theory, this implies that the amino acid *K* at site 200 is most strongly linked to the amino acid *G* at site 202, while their interactions with position 201 with amino acid T are comparatively weak. Decreasing the threshold leads to the extraction of another motif $^{38}SXXK^{41}$ (also appears in Polarity at 0.85 ). In this motif, the sites $38-41$ are strongly linked (the other intermediate positions 39 and 40 with varying amino acids *X* are weakly linked). Two positions 97 and 99 of the third motif $^{97}SDN^{99}$ appear at the 0.9 threshold (appears in Polarizability and Volume at 0.85 threshold ) which becomes the complete SDN motif at 0.85 with the addition of 98. This implies that the sites 97 (*S*) and 99 (*N*) have strong interactions between them while the interactions with position 98 (*D*) are not as strong. There are only three components $38-41$, $200-202$, and $97-98-99$ in the threshold range 0.9 to 0.78. The robustness of these components against varying thresholds gives the superiority of these positions and motifs over other positions. Most of these positions are the major constituent of the specificity-determining sites that contribute to the active, catalytic, and ligand binding sites. The decrease in threshold to 0.75 results in the addition of new components, along with $133-137$ identified as the $^{133}EXXLN^{137}$ motif. Although there is considerable overlap between interaction patterns (and the positions with non-zero links) at low thresholds for different properties but there is a significant divergence at intermediate and high thresholds, indicating unique information revealed by each property. Furthermore, the patterns observed are property-dependent, allowing for the identification of evolutionary interactions between positions for a specific property. The components are shown in Fig. 2 and contributing nodes are given in Supplementary Material Table 3. Most of the positions are crucial for the family as shown by literature studies^35–38^, and many of these positions may hold the potential for acting as targets for future inhibitor design and antibiotic development.

### Multi-link and multi-adjacency

In a multiplex network, we can define multi-links^15,16^ by considering a vector $\vec {m} = (m^1 ,m^2, \ldots , m^{\alpha }, \ldots , m^L )$ where each element $m^{\alpha }$ can takes either either the value 0 or 1. A multi-link between two nodes is defined by the vector $\vec {m}$ such that $m^{\alpha } = 1$ if the two nodes are connected by a link in layer $\alpha$ and zero otherwise. In general, a multi-link between two nodes say i and j is given using the layer adjacency matrix as $\vec {m} = \vec {m_{ij}} = ( a^1_{ij}, a^2_{ij}, \ldots , a^{\alpha }_{ij}, \ldots , a^L_{ij} )$, where $a^{\alpha }$ being the adjacency matrix for network layer $\alpha$. If two given nodes are connected in every layer then $\vec {m} = \vec {1}$ whereas multi-link $\vec {m} = \vec {0}$ signifies that the nodes are not directly connected in any layer of the multiplex network. For *L* layers there are $2^L$ possible multi-links.

Using multi-links, we define the multi-Adjacency matrix^15,16^ as $\mathcal {A}^{\vec {m}}$ with the element $\mathcal {a}_{ij}^{\vec {m}}$ as 1, when there exists a multi-link $\vec {m}$ between node *i* and *j* and 0 other wise. Mathematically, the element $\mathcal {A}_{ij}^{\vec {m}}$ of multi-Adjacency matrix are given in terms of the adjacency matrices $a^{\alpha }$ of layers as

3 $$\begin{aligned} \mathcal {A}_{ij}^{\vec {m}} = \prod _{\alpha =1}^{L} \left[ a_{ij}^{\alpha }m^{\alpha } +(1-a_{ij}^{\alpha })(1-m^{\alpha }) \right] \end{aligned}$$

Due to the normalization condition $\sum\nolimits_{\vec {m}}\mathcal {A}_{ij}^{\vec {m}} =1$, only $2^{L-1}$ out of total of $2^L$ adjacency matrix (one for each $\vec {m}$) are independent. The normalization condition $\sum\nolimits_{\vec {m}}\mathcal {A}_{ij}^{\vec {m}} =1$ implies that if a pair of nodes have one type of multi-link it cannot have another multi-link. The total number of multi-links in a network is equal to the total number of interactions among all pairs of nodes in the network. With multi-adjacency matrix, one can define the *multi-degree* $\mathcal {K}_{i}^{\vec {m}}$ of a node i as $\mathcal {K}_{i}^{\vec {m}} = \sum\nolimits_{j=1}^N \mathcal {A}_{ij}^{\vec {m}}$. Multi-degree of a node represents the number of multi-links connected to that particular node. For example, a network with only two layers ($L=2$), the degree $\mathcal {K}_i^{(1,1)}$ corresponding to $\vec {m} =(1,1)$ is given by $\mathcal {K}_i^{(1,1)} = \sum\nolimits_j a_{ij}^1 a_{ij}^2$. This value indicates the total number of nodes that are simultaneously connected to node *i* in both layers 1 and layer 2.

In the context of the class-A $\beta$-lactamase multiplex network, there are four layers arranged in order of significance: polarity (0001), volume (0010), polarizability (0100), and hydrophobicity (1000). When referring to a specific multi-degree value, such as 1000, it indicates links that are exclusively present in the hydrophobicity layer. On the other hand, a multi-degree of 1001 suggests links that are common to both the hydrophobicity and polarity layers. The multi-degree of each node at the different thresholds for class-A $\beta$-lactamase family is shown in Supplementary Material Figures 5–7. For the class-A $\beta$-lactamase family, at 0.1 thresholds, many pairs of nodes are connected across all network layers simultaneously. These nodes exhibit a non-zero multi-degree value, denoted as $\mathcal {K}_{i}^{\vec {m}}$, where $\vec {m} =(1,1,1,1)$. Out of the total 248 positions, 233 positions have non-zero values for the multi-degree $\mathcal {K}{i}^{1111}$. The presence of a non-zero multidegree $\mathcal {K}_{i}^{1111}$ for a considerable number of nodes at low threshold values can be attributed to statistical noise. With an increase in the threshold, the noise in the system becomes filtered, resulting in fewer pairs of connected nodes in each layer. For instance, at 0.5 threshold, there are only five nodes 46, 124, 137, 156, 199 [**AN: 78, 157, 170, 189, 233**] with a non-zero multidegree $\mathcal {K}_i^{(1111)}$. This indicates that these positions interact in every layer of the network. Notably, positions 124, 137, and 199 are critical residues for catalytic action^38,39^, position 46 shows high conservation ($96\%$) for Gram-negative bacteria^38^. Moreover, certain substitutions at position 156 are known to abolish TEM-1 $\beta$-lactamase activity^40^. However, at a threshold of 0.6, only two nodes 199 and 124 (both significant catalytic residues^38^ ) remain connected in each layer, both having a multidegree of 1, i.e., $\mathcal {K}_i^{(1111)} = 1$, while all other nodes have a multidegree 1 is 0. As we further increase the threshold, no nodes display a non-zero multidegree $\mathcal {K}_i^{(1111)}$.

In class-A $\beta$-lactamase, the omega loop (positions 128-146 with **AN: 161-179**) plays a crucial role in substrate recognition and catalysis^39^. Mutations in this loop can affect the enzyme’s adaptation to new antibiotics. In our analysis at 0.5 threshold, we identified 103 positions that have non-zero multi-degree. Among these, 12 positions fall within the omega loop, that have n0n-zero multi-degree and serve specific functions. Among these, positions 128 and 134 are linked to substrate specificity^36^, Positions 129, 131, 133, 136, 137, and 146 are involved in catalytic and/or substrate binding^38,39^. The remaining positions 130, 142, 143, and 144 are conserved across class-A $\beta$-lactamases^38^. Position 134 which influences substrate activity is active mainly in the polarity layer whereas position 128 interacts more in the polarizability layer. The other catalytic and conserved positions show higher contributions in the hydrophobicity layer or its combination with other properties [Supplementary Material Figures 5–7]. If we further lower the threshold to 0.4, all positions in the omega loop have nonzero multi-degree, except for three positions 132, 138, and 140 [Supplementary Material Table 3].

The quantity $\mathcal {K}_{i}^{\vec {m}}$ serves as a local measure of the link overlap between layers in the multiplex network. It also indicates the strength of interaction between positions within each property layer. For instance, if a position exhibits higher values of $\mathcal {K}_i^{(1000)}$ compared to $\mathcal {K}_i^{(0001)}$, it signifies a greater involvement of that position in hydrophobic interactions rather than polar interactions. The multidegree can establish a hierarchy in the influence of physiochemical properties at a given position. One of the conserved motifs of class-A $\beta$-lactamase family, $^{97}SDN^{99}$ [Supplementary Material Figures 5–7], positions 97, 98, and 99 exhibit varying multidegrees when considering different physiochemical properties represented as distinct layers in the multiplex network analysis. In the hydrophobicity layer (layer 1000), positions 97, 98, and 99 display multidegrees of 1, 1, and 2, respectively. However, when examining the polarizability layer (layer 0100), only position 97 demonstrates a multidegree of 1, while the other two positions have a multidegree of zero. As for the volume layer (layer 0010) and the polarity layer (layer 0001), none of the positions in the conserved motif exhibit a nonzero multidegree. This observation highlights the dominance of hydrophobicity in influencing the positions and the conserved motif. It further suggests that the other properties, with the exception of hydrophobicity (and polarizability for position 97), do not significantly influence these specific positions. Hence, the multi-layer structure of the multiplex network establishes a hierarchy in terms of the influence exerted by each property on individual positions within the class-A $\beta$-lactamase family.

**Figure 3:**
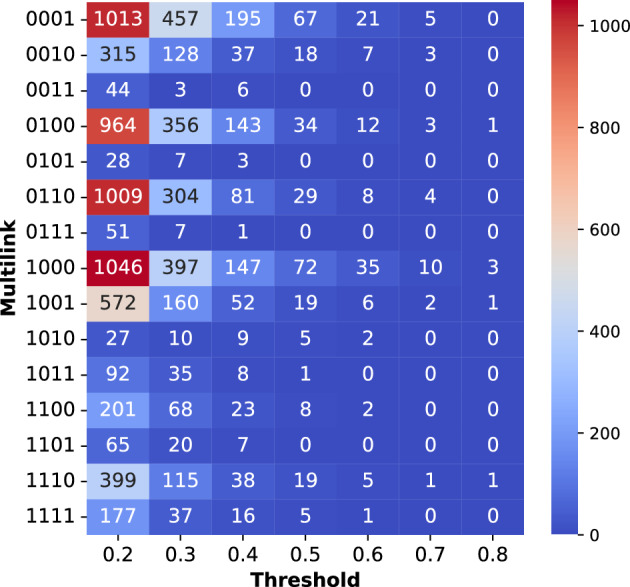
Distribution of multi-links in the network at the different thresholds. The properties are represented from least significant bit to most significant in order of Polarity, Volume, Polarizability, and hydrophobicity.

Figure 3 displays the distribution of multi-links in the multiplex network at various thresholds. The analysis reveals interesting patterns among different property layers. At low and intermediate thresholds ($\theta < 0.5$), Polarity (0001) consistently exhibits the highest number of unique evolutionary links, indicating exclusive connections between specific nodes in this layer compared to others. It is followed by hydrophobicity (1000) and polarizability (0100) with a substantial number of unique links. Conversely, the volume layer (0010) demonstrates the lowest number of unique links. However, at higher thresholds ($\theta > 0.5$), hydrophobicity surpasses polarity in terms of unique links, suggesting a stronger overall evolutionary interaction within the hydrophobicity compared to polarity.

To investigate the collective behavior of property combinations, we examine the simultaneous connections present in different layer combinations. The combination of polarizability and volume (0110) exhibits the highest number of simultaneous connections present in both layers. This is followed by the combinations of hydrophobicity-polarity (1001) and hydrophobicity-polarizability (1100). There are two possible explanations for this observation. First, the two layers may encode similar information (statistical noise), resulting in a higher similarity between the links present in both layers. Alternatively, the two positions may be evolutionarily connected, constituting a functional or structural motif where the properties play vital roles. In such cases, any evolutionary change in one position would affect both properties simultaneously, while leaving other properties unaffected. On the other hand, the combinations of layers (0011, 0101, 1010) exhibit almost zero corresponding multidegrees at intermediate and higher thresholds ($\theta > 0.4$). This suggests that these property combinations have little impact on the evolutionary interaction between positions.

Remarkably, the combination of hydrophobicity, polarizability, and volume (1110) reveals links that are common to all three layers, but absent in the polarity layer. These connections survive even at a very high threshold of 0.8, indicating evolutionary conservation. The presence of these three properties may have an evolutionary impact on protein sequences by potentially imposing functional or structural constraints. In contrast, the other combinations of three properties (0111, 1011, and 1101) exhibit considerably fewer links compared to 1110 at intermediate and higher thresholds. The combination of properties in 1110 (hydrophobicity, polarizability, and volume ) is particularly crucial for the $\beta$-lactamase family. At higher thresholds ($\theta >0.4$) several key positions stand out by showing a high value of multi-degree. For instance at 0.6 threshold for the hydrophobicity layer (1000), the identified positions 97 and 200 play role in substrate binding and catalytic process^38^, position 123, 124, 146, 149, are catalytic positions^38^ and position 208 has an impact cephalosporin resistance^37^. In the polarity layer (0001), the position 187 interacts with ceftazidime and cefotaxime^36^ along with positions 157, 165, 173, and 177 being conserved^38^. Our analysis also uncovers that distinct properties and their combinations highlight different interacting positions. These properties, organized as layers, exhibit varying degrees of influence on evolutionary interactions within the multiplex network. We find that the majority of interacting positions are in the hydrophobicity layer, followed by polarity, polarizability, and, finally, volume (Figures 5–7 in Supplementary Material). This observation implies that hydrophobicity has the most substantial influence on evolutionary interactions, while volume has the least impact. This hierarchy provides a ranking of physicochemical properties in determining evolutionary interactions and positions within the family. The multi-layer structure of the multiplex network provides valuable insights into the hierarchy of physiochemical properties influencing individual positions within the class-A $\beta$-lactamase family, facilitating a targeted approach for understanding and manipulating enzymatic activity to combat antibiotic resistance. This hierarchical ranking of the properties is possible by considering the multiplex structure of the enzyme family.

For any two layers ($\alpha$ and $\gamma$ ) of the network, we can define also a global measure of overlap called **link overlap**, which measures the ratio of the common links in both layers. **Link overlap** quantifies the links connecting the same pair of nodes in both layers and is defined as

4 $$\begin{aligned} \mathcal {O}^{\alpha ,\gamma } = \frac{2\sum_{i=1}^{N}\sum_{j>i}^{N}a_{ij}^{\alpha }a_{ij}^{\gamma }}{\sum_{i=1}^{N}\sum_{j>i}^{N} a_{ij}^{\alpha } + \sum_{i=1}^{N}\sum_{j>i}^{N}a_{ij}^{\gamma }} \end{aligned}$$

**Figure 4:**
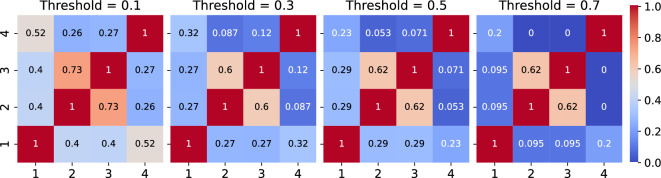
Link overlap between different layers of the multiplex network at the different thresholds. The layers display the physiochemical properties in order 1-hydrophobicity, 2-polarizability, 3-volume, and 4-polarity.

The link overlap between 4 layers of the multiplex network is shown in Fig. 4. At a low threshold ($\theta =0.1$), there is a significant overlap in the interaction among all properties. Specifically, polarizability (represented by 2) and volume (represented by 3) exhibit a remarkably high overlap of over 70%. As the threshold increases, the overlap between layers in the network starts to decrease, although polarizability and volume continue to show significant overlap. As discussed previously, the threshold range $0.0 - 0.2$ represents a noisy region mostly plagued by randomness. The high overlap in this region can be attributed to the random links between positions but these links are very weak in terms of their strength. In the threshold region where the information is predominant ($\theta > 0.3$), there is a sudden decrease in the overlaps and only the actual link contributes. For example, the overlap between polarity (property 4), with volume and polarizability is over 25% at threshold 0.1 but reduces to almost zero at threshold 0.7. Volume and polarizability consistently exhibit an high overlap of approximately 60% across all thresholds, indicating that they encode similar information. Consequently, it will be reasonably safe to reduce the size of the multiplex network by eliminating one of the two layers (either volume or polarizability) with minimal loss of information.

### Multi-strength

Each network layer $G^{\alpha }$ can be independently represented using an adjacency matrix $a^{\alpha }$ for physiochemical property $\alpha$ with $a^{\alpha }_{ij} =1$, if $E^{\alpha }_{i,j} > 0$ and 0 otherwise. Additionally, a weight matrix $w^{\alpha }$ can be defined for property $\alpha$ with $w^{\alpha }_{ij} =E^{\alpha }_{i,j}$, if nodes *i* and *j* are connected and zero otherwise. In weighted networks, to check the heterogeneity in the distribution of weights across the edges, local parameters such as strength are used^15^. The strength of a node *i* in layer $\alpha$ is defined as $\mathcal {S}^{\alpha }_{i} = \sum\nolimits_{j=1}^N w_{ij}^{\alpha }$, which is the sum of weights of all the links incident upon that node.

**Figure 5:**
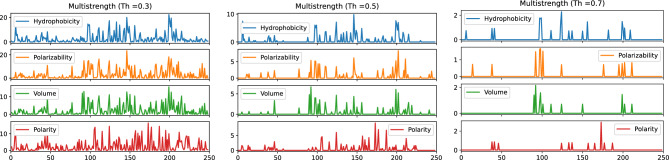
Strength of nodes for multiplex network layers at three different threshold (Th = 0.3, 0.5, 0.7).

Figure 5 reveals important insights about the influence strength of positions in the multiplex network layers. At a low threshold ($\theta = 0.3$), almost all positions exhibit non-zero influence strength. However, as the threshold increases, only a subset of positions retains non-zero influence strength, indicating their significance in the network. Each multiplex layer exhibits distinct nodes with significant influence strength, and these differences become more pronounced at higher thresholds.

At a threshold of 0.7, a few select positions in the network have non-zero influence strength for at least one property. These positions are 6, 14, 38, 41, 46, 90, 92, 97, 98, 99, 101, 111, 123, 124, 133, 137, 150, 156, 157, 165, 173, 176, 177, 182, 187, 195, 199, 200, 202, 209, 211 [**AN: 37, 45, 70, 73, 78, 123, 125, 130, 131, 132, 134, 144, 156, 157, 166, 170, 183, 189, 190, 199, 207, 210, 211, 216, 221, 229, 233, 234, 236, 245, 247**] [Supplementary Material Figure 8]. However, a selected few positions 38, 41, 46, 92, 97, 98, 101, 111, 124, 133, 137, 150, 199, 200, 202, and 211 exhibit non-zero values for at least two properties simultaneously, suggesting their potential relevance and distinct characteristics. Positions 6, 14, 38, 41, 97, 98, 99, 101, 111, 123, 124, 133, 137, 199, 200, 202, 211 are the catalytic positions and/or can influence the enzyme activity^36–38,40^ and the postions 6, 14, 46, 90, 92, 150, 157, 165, 173, 176, 182, 195, 202, 209 are conserved positions^36,38^ Furthermore, certain positions stand out in terms of individual property values. For example, position 124 has the highest strength of 2.28 for hydrophobicity, position 98 demonstrates the highest strength of 1.66 for polarizability, position 92 exhibits the highest strength of 2.16 for volume, and position 182 demonstrates the highest strength of 0.86 for polarity. Notably, a subset of positions (38, 46, 124, 137, 199) displays non-zero values of influence strength for all four properties at 0.6 thresholds [Supplementary Material Figures 8, 9], indicating their simultaneous importance across multiple aspects. The analysis portrays the influence of positions in the multiplex network layers, shedding light on their critical roles and highlighting potential targets for further investigation and modulation of enzymatic activity.

## Conclusions

The use of a multiplex network to analyze the evolutionary interactions between protein sequences yields unparalleled insights into the structural and functional motifs of the class-A $\beta$-lactamase family. By deriving each layer of the network from a correlation matrix calculated using physiochemical properties, we unveil novel information about the intricate interactions between nodes, allowing us to selectively determine key positions and interactions that may act as potential targets for influencing enzymatic or catalytic activity. Although each layer is derived from the same MSA, but it unravels a piece of different information in terms of interaction between the nodes giving useful insight into the functionality and structure of the protein family. We also observe that interaction between the positions depends on the physiochemical properties where positions tend to cluster into groups with identical signs of the property. Our methodology also reveals the hierarchy in the influence of physiochemical properties at a given position, pinpointing the most relevant property responsible for the protein’s functionality. Link overlap analysis reveals that there are limited information exchanges between any two layers, indicating the importance of combining layers to shed light on their collective behavior. The combination of hydrophobicity, polarizability, and volume exhibits common links across all layers, suggesting functional or structural constraints for the class-A $\beta$-lactamase family. In conclusion, the application of a multiplex network provides valuable insights into the function and structure of the class-A $\beta$-lactamase protein family, in identifying key positions, interactions as well as combinations of physiochemical properties. Furthermore, the multiplex network proposed in this study exhibits considerable potential for broader utilization across various protein families, offering invaluable insights into their structural and functional characteristics. This novel technique represents a potent approach for investigating the impact of diverse properties on protein functionality, effectively elucidating crucial motifs and unveiling the intricate hierarchical connections between properties and evolutionary constraints that govern these protein families.

### Supplementary Information

Supplementary Information.

## Data Availability

The datasets analyzed during the study are publicly accessible and can be obtained from the author (P.B.) at reasonable request.
