# Supplementary material for: Exploring complexity of class-A Beta-lactamase family using physiochemical-based multiplex networks
Source: Sci Rep. 2023 Nov 23;13:20626. doi: 10.1038/s41598-023-48128-y (PMC10667273; doi:10.1038/s41598-023-48128-y)
Supplement: Supplementary file 1 — Supplementary Information. [file 41598_2023_48128_MOESM1_ESM.pdf]

Supplementary Material

Exploring Complexity of Class-A Beta-Lactamase family using Physiochemical-Based Multiplex Networks

Data Processing

In the context of multiple sequence alignment (MSA), highly similar sequences can introduce unwanted noise, such as historical and phylogenetic factors, affecting co-evolution analysis. To mitigate this noise, we first refine the MSA by removing sequences with a similarity greater than 90 percent. Identical sequences can also lead to an overrepresentation of patterns, undermining the ability to statistically detect significant correlations. Our approach involves filtering to reduce dataset redundancy, prioritizing a diverse set of sequences without sacrificing essential information. Filtering out highly similar sequences aims to increase sensitivity to genuine co-evolution signals, particularly those arising from functional or structural interactions, thereby enhancing our understanding of the relationships between physico-chemical properties in $\beta-$lactamases. Prior studies have indicated that the optimal similarity range to mitigate phylogenetic effects lies between 70-90%. The proposed method was tested within this range, and for the class-A $\beta-$lactamase family, it proved influential, yielding nearly identical results in the 80-90% similarity range.

After removing sequences with more than 90% similarity, the dataset comprises 559 unique sequences. It is important to note that every protein inherently possesses unique regions within its primary sequence, which are essential for its specific functionality but are not shared among all members of the protein family. As a result, when constructing MSA, gaps are introduced to represent amino acid segments present in one sequence but absent in others. This reflects the dynamic nature of evolution, where amino acids may have been added or removed over time. Numerous gaps within an MSA column may indicate highly variable regions, potentially due to insertions or deletions (indels) that can disrupt alignment and generate misleading co-evolution signals. By eliminating these columns, the focus shifts to conserved regions, which offer more biologically meaningful co-evolution insights while ensuring the analysis remains unaffected by gaps. Columns with gaps exceeding 20 percent are excluded from the MSA, as these positions may lack significant biological relevance, being specific to individual protein sequences rather than the entire protein family.

We used four distinct physiochemical properties to be integrated with MSA. These properties were first extracted from various sources and then re-scaled to a range of -1 to 1, where a value of 1 indicates the highest value of a specific property among all amino acids, while -1 represents the lowest value of the same property. Each amino acid in the MSA is replaced by its rescaled physio-chemical property value. Each physio-chemical property gives a data matrix of the same dimension as MSA by replacing the amino acid with the specific physio-chemical property. Thus a two-dimensional MSA is converted into a three-dimensional data matrix denoted as $D^{\alpha}_{s}(i)$ where α $(= 1 \cdots 4)$ denotes the 4 different physiochemical properties, i represents the column (position of amino acids) in MSA and s represents the row in MSA (protein sequence). For class-A $\beta-$ Lactamase family, each property gives a data matrix D^{α} with dimensions of $S \times L$ (S rows and L columns, where S =559 as the number of sequences and L=248 as columns in MSA). In this way, the two-dimensional MSA ($S \times L$) is now represented as a three-dimensional data matrix ($\alpha \times S \times L$). Notably, the data matrix changes with properties, even though it is derived from the same MSA, suggesting that each property provides unique and valuable information.

The physiochemical property for each amino acid is used and then rescaled between between -1 to 1 (details of physiochemical properties used for analysis are included in the supplementary materials). Each amino acid in the MSA is replaced by its rescaled physiochemical property value. The MSA is replaced by a 3-dimensional data matrix $D^{\alpha}_{s,i}$, where i represents the position (column), s represents the sequence (row) in MSA and α symbolizes the physiochemical property under consideration.

Variation in Network with different number of sequences

Table 1: Variation in the contributing positions at threshold 0.8 for all properties with the number of sequences considered for analysis. Only the interacting positions are listed in the table.

**No. of seq.**	**Hydrophobicity**	**Polarizability**	**Volume**	**Polarity**
**100**	22, 41, 38, 34, 49, 103, 99, 97, 111, 123, 200, 201, 202	98, 97, 211, 202, 201	98, 97, 202, 192	34, 15, 49, 105, 187, 106, 137, 133
**150**	41, 38, 110, 99, 98, 97, 150, 123, 199, 156, 200, 202	199, 92, 110, 98, 97, 195, 176	110, 98, 97	41, 38, 137, 133, 200, 202
**200**	41, 38, 99, 98, 97, 182, 209, 200, 202	98, 101, 97, 211, 202, 201	98, 97	41, 38, 165, 177, 173
**250**	41, 38, 99, 98, 97, 156, 124, 200, 202	98, 97	98, 97	41, 38, 137, 133
**300**	41, 38, 99, 98, 97, 200, 202	98, 101, 97, 211, 202	103, 98, 101, 97	41, 38, 165, 157, 187, 173, 200, 202
**400**	41, 38, 99, 98, 97, 200, 202	98, 97, 211, 202	98, 97	41, 38
**500**	41, 38, 99, 98, 97, 200, 202	98, 97, 211, 202	98, 97	41, 38

Table 2: Variation in Topological Properties of the class-A $\beta-$lactamase family such as number of edges, average clustering (C_{avg}), average degree (K_{avg}), maximum degree (K_{max}), size of largest component (N_{comp}) at 0.8 threshold variation in the Topological properties of network layers at threshold 0.8 with the number of sequences (No. of Seq.) used for analysis.

**Property**	**No. of Seq**	**Edges**	C_{avg}	K_{avg}	K_{max}	N_{comp}
Hydrophobicity	100	10	0.024	0.081	2	3
	200	6	0.012	0.048	2	3
	300	5	0.012	0.040	2	3
	400	4	0.000	0.032	2	3
	500	5	0.012	0.040	2	3
Polarizability	100	3	0.000	0.024	2	3
	200	4	0.000	0.032	2	3
	300	4	0.012	0.032	2	3
	400	2	0.000	0.016	1	2
	500	2	0.000	0.016	1	2
Volume	100	2	0.000	0.016	1	2
	200	1	0.000	0.008	1	2
	300	3	0.000	0.024	2	4
	400	1	0.000	0.008	1	2
	500	1	0.000	0.008	1	2
Polarity	100	5	0.000	0.040	2	3
	200	3	0.000	0.024	2	3
	300	6	0.009	0.048	3	4
	400	1	0.000	0.008	1	2
	500	1	0.000	0.008	1	2

Positions with non-zero interactions

Table 3: Positions with the non-zero interactions for different properties at varying threshold (θ)

θ	**Hydrophobicity**	**Polarizability**	**Volume**	**Polarity**
**0.4**	5, 6, 7, 8, 9, 10, 14, 19, 20, 22, 23, 29, 31, 32, 34, 38, 39, 40, 41, 42, 43, 44, 46, 49, 52, 53, 57, 58, 59, 63, 85, 88, 89, 90, 92, 97, 98, 99, 103, 104, 108, 110, 111, 116, 123, 124, 127, 130, 131, 133, 135, 137, 142, 143, 146, 147, 148, 149, 150, 156, 157, 162, 163, 165, 169, 172, 173, 176, 177, 182, 183, 186, 188, 190, 192, 194, 195, 199, 200, 201, 202, 203, 204, 205, 206, 208, 209, 213, 214, 215, 218, 221, 225, 227, 230, 234, 239, 240, 247	6, 9, 11, 12, 14, 16, 26, 29, 33, 35, 36, 38, 39, 42, 45, 46, 55, 65, 74, 77, 90, 92, 93, 95, 97, 98, 99, 100, 101, 102, 103, 110, 111, 112, 114, 118, 120, 123, 124, 127, 128, 130, 131, 133, 134, 137, 142, 143, 144, 145, 146, 147, 148, 149, 150, 152, 156, 163, 164, 169, 170, 176, 180, 182, 183, 187, 188, 189, 190, 192, 194, 195, 197, 198, 199, 200, 201, 202, 203, 204, 205, 206, 208, 209, 211, 214, 215, 219, 223, 224, 226, 228, 230, 233, 238, 240, 241, 244	5, 9, 11, 12, 13, 14, 16, 29, 35, 38, 39, 42, 43, 46, 55, 75, 77, 89, 90, 92, 95, 97, 98, 99, 101, 102, 103, 105, 109, 110, 111, 114, 118, 123, 124, 128, 129, 130, 131, 134, 137, 141, 142, 144, 145, 146, 147, 150, 152, 156, 162, 165, 169, 170, 173, 176, 177, 180, 182, 183, 189, 190, 192, 195, 198, 199, 200, 201, 202, 203, 205, 207, 208, 209, 211, 214, 215, 219, 223, 224, 226, 228, 230, 238, 241, 244	1, 4, 5, 6, 9, 13, 15, 19, 20, 32, 34, 36, 37, 38, 39, 40, 41, 42, 43, 45, 46, 48, 49, 52, 53, 57, 58, 59, 63, 65, 80, 84, 86, 89, 93, 94, 98, 99, 102, 103, 104, 105, 106, 108, 109, 111, 115, 118, 119, 123, 124, 126, 127, 129, 130, 131, 133, 136, 137, 139, 143, 146, 147, 150, 153, 156, 157, 160, 161, 162, 164, 165, 169, 173, 176, 177, 180, 182, 186, 187, 191, 195, 197, 199, 200, 204, 208, 209, 210, 211, 212, 213, 214, 215, 219, 220, 221, 222, 224, 225, 230, 236, 237, 240, 245
**0.5**	6, 7, 8, 14, 20, 22, 34, 38, 39, 41, 46, 49, 57, 88, 97, 98, 99, 103, 110, 111, 116, 123, 124, 127, 130, 133, 137, 142, 143, 146, 147, 148, 149, 150, 156, 157, 162, 163, 165, 173, 177, 182, 183, 186, 194, 195, 199, 200, 201, 202, 203, 205, 208, 209, 214, 215, 230	11, 12, 14, 29, 38, 39, 46, 77, 90, 92, 97, 98, 101, 103, 110, 111, 123, 124, 128, 131, 133, 137, 144, 146, 147, 148, 149, 152, 156, 176, 182, 183, 189, 192, 195, 198, 199, 201, 202, 203, 209, 211, 214, 230, 241, 244	5, 14, 29, 38, 46, 77, 90, 92, 97, 98, 101, 103, 110, 111, 118, 123, 124, 128, 129, 134, 137, 142, 146, 147, 150, 152, 156, 176, 183, 189, 192, 195, 198, 199, 202, 203, 208, 209, 211, 214, 215, 219, 230, 241	5, 6, 13, 15, 34, 38, 39, 41, 43, 45, 46, 49, 57, 89, 94, 98, 99, 105, 106, 115, 124, 130, 133, 136, 137, 143, 146, 153, 156, 157, 160, 162, 165, 173, 176, 177, 180, 182, 186, 187, 191, 195, 197, 199, 209, 212, 213, 215, 220, 221, 222, 224, 225
**0.6**	6, 14, 38, 41, 46, 97, 98, 99, 103, 110, 111, 123, 124, 133, 137, 142, 143, 146, 147, 149, 150, 156, 182, 183, 194, 199, 200, 201, 202, 203, 208, 209, 215	14, 38, 46, 77, 92, 97, 98, 101, 103, 110, 111, 124, 133, 144, 146, 147, 176, 195, 198, 199, 201, 202, 203, 209, 211, 241	14, 38, 46, 90, 92, 97, 98, 101, 103, 110, 111, 124, 137, 150, 156, 176, 192, 195, 199, 202, 211, 215	6, 13, 34, 38, 41, 46, 105, 106, 115, 124, 133, 137, 156, 157, 160, 165, 173, 177, 182, 187, 199, 209, 212
**0.7**	6, 38, 41, 97, 98, 99, 111, 123, 124, 133, 137, 150, 156, 182, 199, 200, 202, 209	14, 38, 92, 97, 98, 101, 124, 176, 195, 199, 202, 211	90, 92, 97, 98, 101, 111, 124, 150, 199, 202, 211	38, 41, 46, 124, 133, 137, 157, 165, 173, 177, 187
**0.8**	38, 41, 97, 98, 99, 200, 202	97, 98, 202, 211	97, 98	38, 41

Positions with non-zero degree at different thresholds.

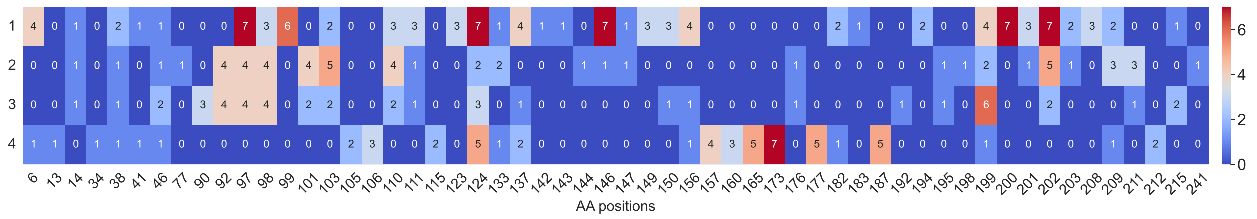

Figure 1: Degree of nodes in each network layer at 0.6 thresholds. Only the nodes with non-zero contributions in at least one network layer are shown for clarity.

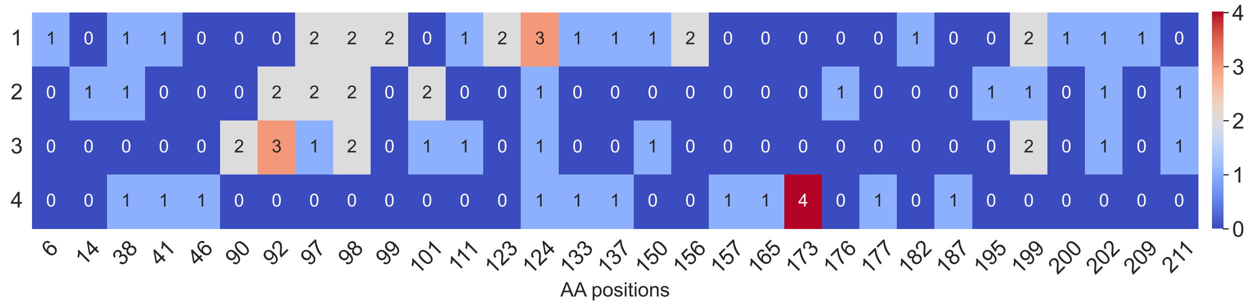

Figure 2: Degree of nodes in each network layer at 0.7 threshold. Only the nodes with non-zero contributions in at least one network layer are shown for clarity.

Positions with non-zero clustering coefficient at different thresholds.

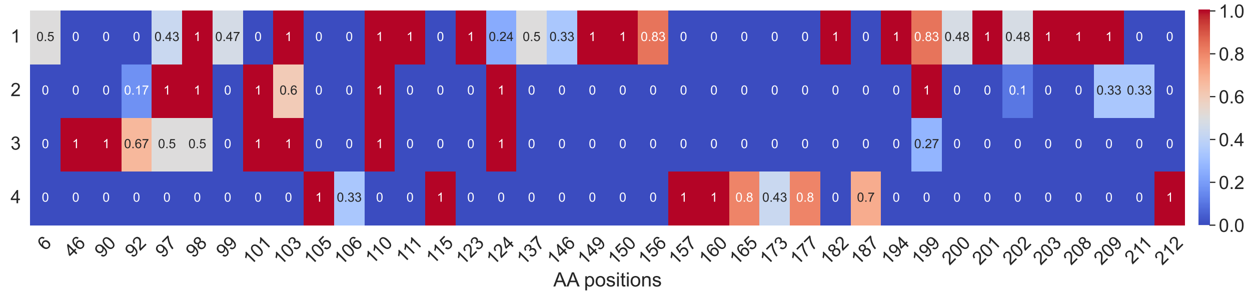

Figure 3: Clustering coefficients of nodes in each network layer at 0.6 thresholds. Only the nodes with non-zero contributions in at least one network layer are shown for clarity.

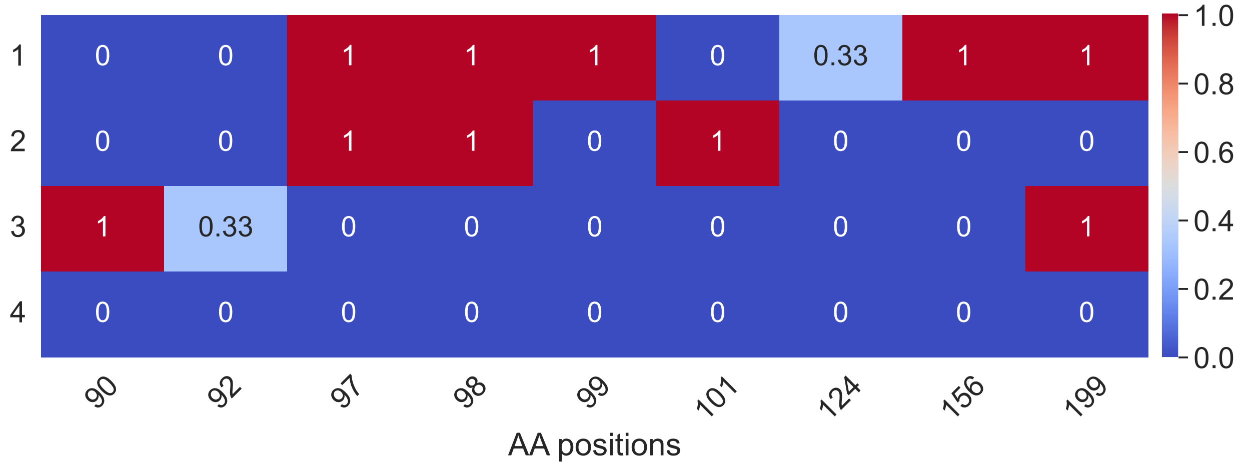

Figure 4: Clustering coefficients of nodes in each network layer at 0.7 thresholds. Only the nodes with non-zero contributions in at least one network layer are shown for clarity.

Positions with non-zero multi-degree at different thresholds.

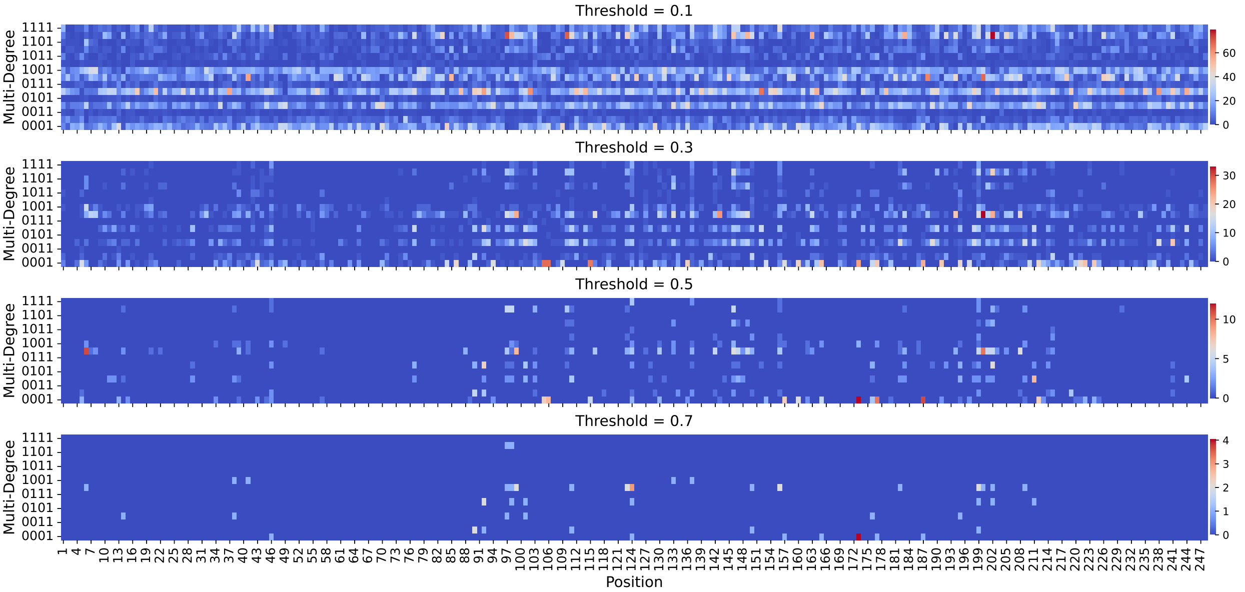

Figure 5: Multi-degree of each node at different thresholds. The properties are represented from least significant bit to most significant in order of Polarity, Volume, Polarizability, and hydrophobicity.

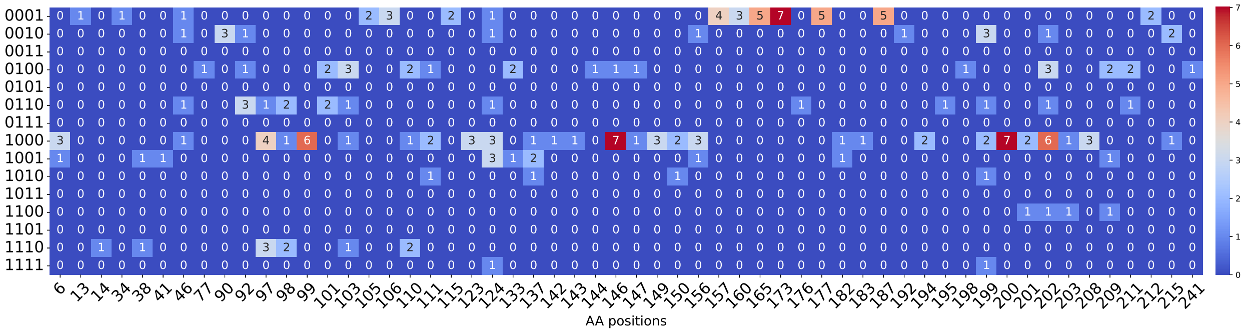

Figure 6: Multidegree of nodes in each network layer at 0.6 thresholds. Only the nodes with non-zero contributions in at least one network layer are shown for clarity.

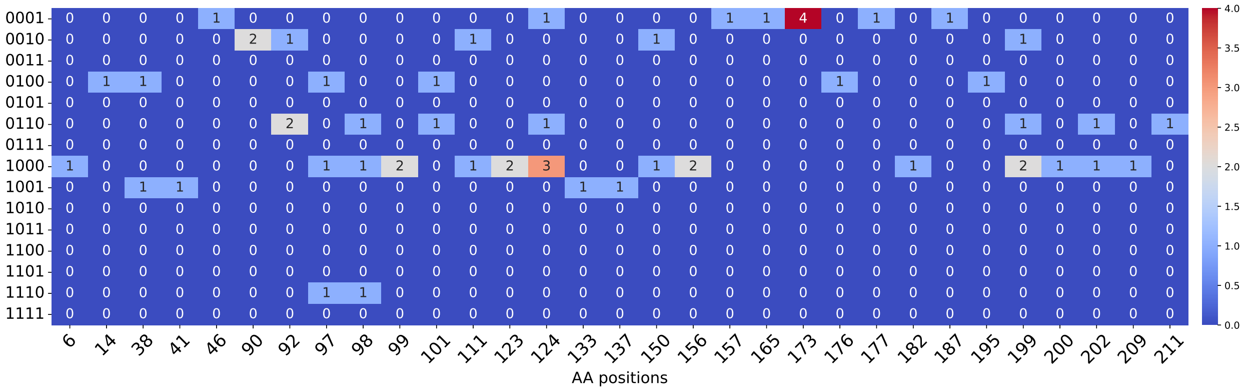

Figure 7: Clustering coefficients of nodes in each network layer at 0.6 thresholds. Only the nodes with non-zero contributions in at least one network layer are shown for clarity.

Positions with non-zero influence strength at different thresholds.

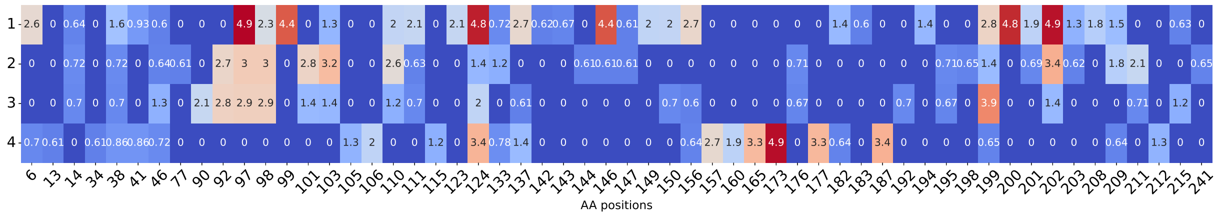

Figure 8: Influence strength of nodes in each network layer at 0.6 thresholds. Only the nodes with non-zero contributions in at least one network layer are shown for clarity.

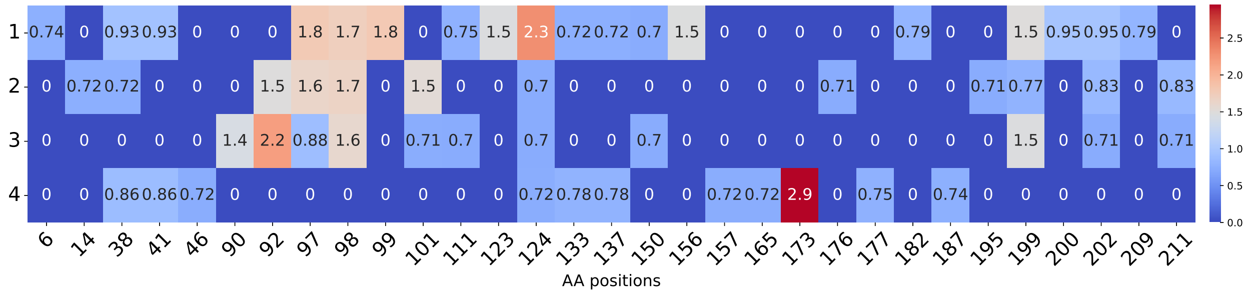

Figure 9: Influence strength of nodes in each network layer at 0.7 thresholds. Only the nodes with non-zero contributions in at least one network layer are shown for clarity.

Details of the four properties used for the construction of multiplex network

Table 4: List of physicochemical properties used for analysis

Amino Acid	Normalized average hydrophobicity	Polarizability parameter	Volume	Polarity
A	0.02	0.046	31	8.1
C	0.77	0.128	55	5.5
D	-1.04	0.105	54	13
E	-1.14	0.151	83	12.3
F	1.35	0.29	132	5.2
G	-0.8	0	3	9
H	0.26	0.23	96	10.4
I	1.81	0.186	111	5.2
K	-0.41	0.219	119	11.3
L	1.14	0.186	111	4.9
M	1	0.221	105	5.7
N	-0.77	0.134	56	11.6
P	-0.09	0.131	32.5	8
Q	-1.1	0.18	85	10.5
R	-0.42	0.291	124	10.5
S	-0.97	0.062	32	9.2
T	-0.77	0.108	61	8.6
V	1.13	0.14	84	5.9
W	1.71	0.409	170	5.4
Y	1.11	0.298	136	6.2

Table 5: Mapping between the Ambler number scheme and the nodes in the multiplex network. The amino acid sequence from 1SHV.pdb is shown just for reference.

Ambler	1SHV	Nodes	Ambler	1SHV	Nodes	Ambler	1SHV	Nodes	Ambler	1SHV	Nodes	Ambler	1SHV	Nodes	Ambler	1SHV	Nodes
1			51	L	20	101	D	69	151	F	118	201	A	167	251	P	215
2			52	A	21	102	L	70	152	L	119	202	R	168	252	N	216
3			53	S	22	103	V	71	153	R	120	203	S	169	253	N	217
4			54	G	23	104	D	72	154	Q	121	204	Q	170	254	K	218
5	M		55	R	24	105	Y	73	155	I	122	205	R	171	255	A	
6	R		56	T	25	106	S	74	156	G	123	206	Q	172	256	E	219
7	Y		57	L	26	107	P	75	157	D	124	207	L	173	257	R	220
8	I		58	T		108	V	76	158	N	125	208	L	174	258	I	221
9	R		59	A	27	109	S	77	159	V	126	209	Q	175	259	V	222
10	L		60	W	28	110	E	78	160	T	127	210	W	176	260	V	
11	C		61	R	29	111	K	79	161	R	128	211	M	177	261	I	223
12	I		62	A	30	112	H	80	162	L	129	212	V	178	262	Y	224
13	I		63	D	31	113	L		163	D	130	213	D	179	263	L	225
14	S		64	E	32	114	A	81	164	R	131	214	D	180	264	R	226
15	L		65	R	33	115	D	82	165	W	132	215	R	181	265	D	227
16	L		66	F	34	116	G	83	166	E	133	216	V	182	266	T	228
17	A		67	P	35	117	M	84	167	T	134	217	A	183	267	P	
18	T		68	M	36	118	T	85	168	E	135	218	G	184	268	A	229
19	L		69	M	37	119	V	86	169	L	136	219	P	185	269	S	
20	P		70	S	38	120	G	87	170	N	137	220	L	186	270	M	230
21	L		71	T	39	121	E	88	171	E	138	221	I	187	271	A	231
22	A		72	F	40	122	L	89	172	A	139	222	R	188	272	E	232
23	V		73	K	41	123	C	90	173	L	140	223	S	189	273	R	233
24	H		74	V	42	124	A	91	174	P	141	224	V	190	274	N	234
25	A		75	V	43	125	A	92	175	G	142	225	L	191	275	Q	235
26	S		76	L	44	126	A	93	176	D	143	226	P	192	276	Q	236
27	P		77	C	45	127	I	94	177	A	144	227	A	193	277	I	237
28	Q		78	G	46	128	T	95	178	R	145	228	G	194	278	A	238
29	P		79	A	47	129	M	96	179	D	146	229	W	195	279	G	239
30	L	1	80	V	48	130	S	97	180	T	147	230	F	196	280	I	240
31	E	2	81	L	49	131	D	98	181	T	148	231	I	197	281	G	241
32	Q	3	82	A	50	132	N	99	182	T	149	232	A	198	282	A	242
33	I	4	83	R	51	133	S	100	183	P	150	233	D	199	283	A	243
34	K		84	V	52	134	A	101	184	A	151	234	K	200	284	L	
35	L		85	D	53	135	A	102	185	S	152	235	T	201	285	I	244
36	S	5	86	A	54	136	N	103	186	M	153	236	G	202	286	E	245
37	E	6	87	G	55	137	L	104	187	A	154	237	A	203	287	H	246
38	S	7	88	D	56	138	L	105	188	A	155	238	G		288	W	247
39	Q	8	89	E	57	139	L	106	189	T	156	239	E		289	Q	248
40	L	9	90	Q	58	140	A	107	190	L	157	240	R	204	290	R	
41	S	10	91	L	59	141	T	108	191	R	158	241	G	205			
42	G	11	92	E	60	142	V	109	192	K	159	242	A	206			
43	R	12	93	R	61	143	G	110	193	L	160	243	R	207			
44	V	13	94	K	62	144	G	111	194	L	161	244	G	208			
45	G	14	95	I	63	145	P	112	195	T	162	245	I	209			
46	M	15	96	H	64	146	A	113	196	S	163	246	V	210			
47	I	16	97	Y	65	147	G	114	197	Q	164	247	A	211			
48	E	17	98	R	66	148	L	115	198	R		248	L	212			
49	M	18	99	Q	67	149	T	116	199	L	165	249	L	213			
50	D	19	100	Q	68	150	A	117	200	S	166	250	G	214			
